# A survey to determine usual care after cancer treatment within the United Kingdom national health service

**DOI:** 10.1186/s12885-017-3172-1

**Published:** 2017-03-11

**Authors:** M. Duncan, J. Deane, P. D. White, D. Ridge, R. Roylance, A. Korszun, T. Chalder, K. S. Bhui, M. A. Thaha, L. Bourke, on behalf of the SURECAN investigators, Steph Taylor, Sandra Eldridge, Paul McCrone, Adrienne Morgan, Louise Jones, Gail Eva, John Gribben

**Affiliations:** 1 Wolfson Institute of Preventive Medicine, Barts and the London School of Medicine and Dentistry, Queen Mary University of London, London, UK; 2 Institute of Health and Society, Newcastle University, Newcastle upon Tyne, UK; 3 Department of Psychology, University of Westminster, London, UK; 4 University College Hospitals London, London, UK; 5 Institute of Psychiatry, King’s College London, London, UK; 6 National Bowel Research Centre, Blizard Institute, Barts and The London School of Medicine and Dentistry, Queen Mary University of London, London, UK; 7 Sheffield Hallam University, Office A121, Collegiate Hall, Collegiate Crescent, Sheffield, S10 2BP UK

**Keywords:** Cancer, Survey, Quality of Life, NHS, Post-treatment, Follow-up

## Abstract

**Background:**

Approximately one third of cancer survivors in the United Kingdom face ongoing and debilitating psychological and physical symptoms related to poor quality of life. Very little is known about current post-cancer treatment services.

**Methods:**

Oncology healthcare professionals (HCPs) were invited to take part in a survey, which gathered both quantitative and free text data about the content and delivery of cancer aftercare and patient needs. Analysis involved descriptive statistics and content analysis.

**Results:**

There were 163 complete responses from 278 survey participants; 70% of NHS acute trusts provided data. HCPs views on patient post-cancer treatment needs were most frequently: fear of recurrence (95%), fatigue (94%), changes in physical capabilities (89%), anxiety (89%) and depression (88%). A median number of 2 aftercare sessions were provided (interquartile range: 1,4) lasting between 30 and 60 min. Usually these were provided face-to-face and intermittently by a HCP. However, sessions did not necessarily address the issues HCPs asserted as important. Themes from free-text responses highlighted inconsistencies in care, uncertain funding for services and omission of some evidence based approaches.

**Conclusion:**

Provision of post-cancer treatment follow-up care is neither universal nor consistent in the NHS, nor does it address needs HCPs identified as most important.

**Electronic supplementary material:**

The online version of this article (doi:10.1186/s12885-017-3172-1) contains supplementary material, which is available to authorized users.

## Background

Two million people now live with or beyond cancer in the UK [1]. Although many cancer survivors report good health, a substantial proportion of between 10 and 20% (those without a chronic condition), may have ongoing poor health and serious disability. For those with an additional chronic condition this may be as high as 25–30% [2]. A national survey (*n* = 3300) assessing the quality of life (QoL) of adult cancer survivors reported that issues affecting cancer survivors included: fear of recurrence (57%), fatigue (43%), body image concerns (31%) complete lack of exercise (30%) and sexual problems (27%) [3]. Prospective cohort data revealed similar findings with 30% of UK cancers survivors reporting more than five unmet needs or problems, including fear of recurrence, fatigue, anxiety, depression, limited independent living and not knowing how to “get better” - the majority of these issues remained unresolved [4]. Consequently, we surmise around a third of NHS cancer survivors have poor QoL related to multiple ongoing and unaddressed problems. Such issues are of course not unique to the UK: both in Europe [5] and the US [6–8] cancer survivorship initiatives and addressing unmet post-treatment needs is being increasingly recognised as an important part of cancer care.

Given the move to patient directed self-management following active cancer treatment, it is essential that individuals with ongoing poor health and related problems are identified and offered appropriate support. No recent studies in the UK have assessed how or the extent to which ongoing unmet needs are identified and what measures are taken to improve or recover post-treatment QoL for cancer survivors within the NHS. Hence, it is currently unclear whether there is a cohesive or system-wide approach to these issues. This is despite an existing evidence base for interventions that can address some of the problems faced by people during and after cancer treatment. For example, psychosocial interventions have been shown to have beneficial effects on depression, anxiety and stress [9, 10]. Cognitive behavioural therapy (CBT) has been shown to benefit and sustain QoL improvements in cancer survivors [11, 12]. Similarly, exercise interventions have received support for benefiting QoL in several meta-analyses [13–15]. Where appropriate, vocational rehabilitation and helping people return to work are also of critical importance from an individual and economic perspective [16, 17]. Therefore, the aim of this survey was to assess service provision for patients completing curative treatment for cancer in UK NHS practice, together with the views of health care professionals (HCPs) about areas for improvement in the current service provision.

## Methods

### Measures

A 22 item standardised survey, including Likert scoring and free text questions, was designed to determine what is provided as part of usual care within the NHS for patients who have finished active cancer treatment with curative intent, it also asked what particular problems were thought to be related to poor QoL (see online Additional file 1). The survey was ’standardised’ in that the same questions were presented in the same format, sequence and via the same delivery method (i.e. online) to all participants. The survey was developed through feedback after piloting it with cancer HCPs and patients. All questions required an answer. The survey was sent to a range of professional bodies to try to capture all relevant HCPs involved in the management of cancer patients working in a range of clinical settings. These included the Association of Cancer Physicians, the UK Oncology Nurses Society, the Royal College of Radiologists, the UK Breast Intergroup, British Psychological Society and the Association of Coloproctology of Great Britain and Ireland. The professional bodies distributed the survey to their members via email link that was open from August to December 2015. The survey was sent to HCPs only. The approval for the survey as a service evaluation was gained via Barts Health NHS trust (Reg No. 6131).

### Respondent characteristics

Participants were asked their country of practice (within the UK), profession, cancer speciality, institute type and number of years of practice in cancer care.

### Service provision

Participants were asked how many people living with and beyond cancer attend their service yearly, whether a specific assessment was employed, an intervention or therapy provided, and which professionals were involved in this provision. A five point Likert scale (never to always) was used to ask respondents whether specific interventions were offered, and what aspects of living beyond cancer were addressed in their service. Five more questions asked respondents about the format, number, frequency and duration of the interventions offered and how the sessions were delivered (e.g. face-to-face, telephone etc).

### Professional opinion

Participants were asked to rate what were the most important needs of people living with and beyond cancer on a 5 point Likert scale (unimportant to very important). A free text box was also provided to allow participants to describe what more could be done to support people living with and beyond cancer.

### Procedure

The survey questions were initially piloted with oncology consultants experienced in the management of a range of malignancies to ensure that the content, language and length of the survey were appropriate. Patient feedback was also incorporated. Following participant feedback, survey questions were refined before being sent out to study participants. The aforementioned professional bodies and groups were contacted and agreed to distribute the final survey to their members via a link in an email. The participants completed the survey through the online tool SurveyMonkey (https://www.surveymonkey.com/). It was made clear to participants that the survey referred to the care of people who have completed their cancer treatment delivered with curative intent.

### Data analysis

The results of the survey were summarised using descriptive statistics (e.g. percentages, means and standard deviations, as appropriate). To aid data interpretation, some responses were converted into categorical scores, for example, the four answer options ‘never’, ‘rarely’, ‘frequently’ and ‘always’ were merged into two categories by collapsing ‘never’ and ‘rarely’ and ‘frequently’ and ‘always’. The free text responses were analysed using thematic analysis [18]. Emerging themes were identified by MD using the Nvivo software to aid the coding and organisation of the free text responses. The initial analysis involved continually moving between reviewing the responses, coding, linking codes, and revising and reshaping the analysis. MD then wrote up thematic topic summaries, which were reviewed by LB and DR.

## Results

### Participants

There was a total of 278 responses, of which 163 had completed through to the end of the survey, resulting in a 59% full completion rate. 108 of the 154 (70%) acute NHS trusts were represented in the survey (i.e. at least one response from an individual within a NHS acute trust). We received responses from the Association of Cancer Physicians, the UK Oncology Nurses Society, the Royal College of Radiologists, and the UK Breast Intergroup. No response was obtained from The British Psychological Society and the Association of Coloproctology of Great Britain and Ireland. We were unable to calculate an absolute response rate as the respective professional bodies acted as intermediaries in the process in order to protect the privacy of their members and handled dissemination of the survey link. The median number of patients attending the HCP’s service that were classed as living with and beyond cancer was 300 (range 0 – 5000). Breast and colo-rectal specialists, from dedicated cancer centres with between 11 and 25 years of practice experience made up the majority of the survey responders. Full characteristics of the survey responders are reported in Table 1.

**Table 1:** Respondent characteristics

Country of service	England	83% [231]
	Scotland	9% [26]
	Wales	4% [12]
	Northern Ireland	3% [9]
Profession	Other^a^	33% [89]
	Medical Oncologists	30% [82]
	Clinical Nurse Specialists	20% [54]
	Clinical Oncologists	7% [20]
	Surgeon	6% [16]
	Medical Oncology trainee	3% [9]
	Psychologist	0% [1]
Cancer specialty◊	Breast	49% [129]
	Colorectal	23% [61]
	Lung	23% [59]
	Other (please specify)^b^	23% [59]
	Gynaecological	22% [58]
	Urological	22% [58]
	Upper gastro-intestinal	17% [45]
	Head and neck	13% [33]
	Sarcoma	13% [33]
	Hepatobiliary system	11% [28]
	Dermatology	9% [23]
	Lymphoma	8% [22]
	Central Nervous System	8% [22]
	Multiple Myeloma	8% [20]
	Leukaemia	5% [14]
Institution◊	Cancer centre	52% [115]
	Teaching hospital	29% [64]
	District hospital	24% [53]
	General hospital	19% [43]
	Other (please specify)^c^	4% [8]
	Community facility	1% [2]
	Primary care	1% [2]
Years practising in cancer care	26+ years	19% [49]
	16–20 years	23% [59]
	11–15 years	24% [64]
	21–25 years	13% [35]
	6–10 years	13% [33]
	0–5 years	8% [22]

^a^Other answers included: radiographer (49%), nurse (12%), research nurses (9%), radiotherapists (6%), centre managers (5%), lay person (3%), survivorship leads (3%), advanced nurse practitioners (2%), dosimetrists (1%), occupational therapists (1%), physiotherapists (1%), radiologists (1%), and research coordinator (1%)

^b^Other answers included: melanoma, primary cancer, all tumour sites, testicular, paediatric, and geriatric

^c^Other answers included: private hospitals, information and support services

◊Multiple options available to respond and will add up to over 100%

### Service provision

Seventy one percent of respondents reported that their unit provided some form of specific assessment, interventions, or therapy for people who have completed active treatment. HCPs identified that clinical nurse specialists (92%), clinical oncologists (62%), medical oncologists (54%), counsellors (51%), physiotherapists (52%), psychologists (45%), surgeons (40%), and occupational therapists (35%) were involved in post-treatment follow-up care.

The five most frequent aspects of post-treatment care specifically addressed as part of follow-up were fatigue (84%), fear of recurrence (83%), anxiety (82%) depression/low mood (78%) and menopausal problems (76%): please see Table 2 for full details. These elements were similar in some respects to the HCP’s top five views of patient needs, these being fear of recurrence (95%), fatigue (94%), changes in physical capabilities (89%), anxiety (89%) and depression (88%). Please see Table 3 for full details. However, the specifics of the dedicated interventions delivered were broadly incongruent to HCPs views on what was important to address in practice. The five most common interventions offered were reported as diet advice (72%), a medical assessment (69%), exercise advice (65%), a one off ‘end of care’ assessment (62%) and counselling (61%). Interventions such as CBT (16%), mindfulness (21%) and return to work support (20%) were amongst the most infrequently offered (please see Table 4). It was not clear how HCP priority areas of fear of recurrence, depression and anxiety were addressed in practice. Interventions provided were most often delivered by HCP on an individual basis (76%), a median of two sessions, lasting 30 min to 1 h (53%). Specific details of the interventions offered including the format, number, duration, frequency and delivery method are described in Table 5.

**Table 2:** Elements of post-cancer treatment addressed in aftercare

Aspects of living beyond cancer addressed			
	% of HCP responding Frequently/Always	% of HCP responding Occasionally	% of HCP responding Rarely/Never
Fatigue	84% [115]	10% [13]	7% [9]
Fear of recurrence	83% [113]	13% [18]	4% [6]
Anxiety	82% [112]	15% [21]	3% [4]
Depression/low mood	78% [107]	19% [26]	3% [4]
Menopausal symptoms if applicable	76% [102]	12% [16]	12% [16]
Financial problems	72% [99]	24% [33]	4% [5]
Body image problems	72% [98]	21% [29]	7% [10]
Long-term medical complications of treatment	72% [98]	20% [27]	8% [11]
Changes in physical capacity	68% [93]	24% [33]	7% [10]
Osteoporosis	66% [89]	20% [27]	15% [20]
Other emotional reactions (e.g. guilt, shame, anger)	65% [87]	28% [37]	7% [10]
Fear of death	63% [86]	28% [38]	9% [12]
Weight changes	56% [77]	34% [46]	10% [14]
Social problems	55% [75]	34% [47]	11% [15]
Low self esteem	53% [72]	31% [42]	16% [21]
Sexual difficulties	48% [66]	34% [47]	18% [24]
Changes in cognitive capacity	45% [62]	34% [47]	21% [28]
Vocational/occupational problems	37% [51]	42% [57]	21% [29]
Spiritual needs	36% [48]	35% [47]	30% [40]

Note: figures in square parentheses are absolute numbers of responders

**Table 3:** HCP’s views of needs of those living with and beyond cancer

	Very important or important	Moderately important	Unimportant or of little importance
Fear of recurrence	95% [174]	4% [7]	1% [2]
Fatigue	94% [172]	5% [9]	1% [2]
Anxiety	89% [163]	10% [18]	1% [2]
Changes in physical capacity	89% [163]	10% [19]	1% [1]
Depression/low mood	88% [161]	11% [20]	1% [2]
Long-term medical complications of treatment	85% [156]	11% [20]	4% [7]
Body image problems	83% [152]	15% [27]	2% [4]
Fear of death	81% [149]	16% [30]	2% [4]
Financial problems	81% [148]	16% [30]	3% [5]
Menopausal symptoms if applicable	79% [145]	17% [31]	4% [7]
Sexual difficulties	79% [144]	18% [32]	4% [7]
Changes in cognitive capacity	77% [143]	21% [38]	3% [5]
Other emotional reactions (eg. guilt, shame, anger)	75% [138]	19% [35]	6% [10]
Social problems	73% [134]	21% [39]	6% [10]
Weight changes	73% [133]	25% [45]	3% [5]
Vocational/occupational problems	72% [131]	20% [37]	8% [15]
Osteoporosis	67% [122]	28% [51]	6% [10]
Low self esteem	66% [118]	28% [52]	6% [10]
Spiritual needs	58% [106]	28% [51]	14% [26]

Note: figures in square parentheses are absolute numbers of responders

**Table 4:** HCPs reports of Interventions offered in aftercare

	Frequently/Always	Occasionally	Rarely/Never	Don’t know
Interventions offered				
Dietary advice or support	72% [105]	19% [28]	5% [7]	4% [6]
Medical assessment	69% [100]	21% [30]	10% [14]	1% [2]
Exercise therapy or advice	65% [95]	18% [26]	11% [16]	6% [9]
One off assessment at the end of treatment	62% [91]	12% [18]	17% [24]	9% [13]
Counselling	61% [89]	30% [44]	6% [9]	3% [4]
Peer Support	57% [83]	25% [36]	8% [12]	10% [15]
Family counselling/therapy	23% [34]	37% [54]	28% [41]	12% [17]
Mindfulness training	21% [30]	20% [29]	31% [45]	29% [42]
Vocational rehabilitation/return to work programme	20% [29]	19%	32% [47]	30% [43]
Cognitive behavioural therapy	16% [24]	40% [59]	25% [36]	19% [27]
Acceptance and commitment therapy	9% [10]	10% [14]	34% [49]	50% [73]

**Table 5:** Delivery of the interventions delivered in after care

*Format of sessions ◊*		
	Individual support facilitated by health care professionals	76% [100]
	Group support facilitated by healthcare professional	74% [97]
	Group peer support	65% [85]
	Individual peer support	17% [22]
	None of the above (please specify)^a^	5% [7]
*Number of sessions of aftercare offered*	1	28% [36]
	2	18% [23]
	3	17% [22]
	4	10% [13]
	5	13% [17]
	More than 5 (please specify number)^b^	15% [20]
*Duration of session*	Less than 30mins	21% [27]
	30 min – 1 h	53% [69]
	1 – 2 h	18% [24]
	2 h +	9% [11]
*Frequency of sessions*	Once	6% [8]
	Weekly	22% [28]
	Fortnightly	6% [7]
	Monthly	10% [13]
	Intermittently	33% [42]
	Not applicable	12% [15]
	Less often (please specify)	11% [14]
*Delivery of sessions ◊*	Face-to-face	95% [121]
	Telephone	37% [47]
	Other (please specify)	8% [10]
	Web based	2% [2]
	Skype/Facetime	0.0% [0]

Note: figures in square parentheses are absolute numbers of responders

^a^Other answers included: in response to need, none known, signposting to 3rd sector services

^b^Other answers ranged from 0 to 99, median = 7.5

◊multiple options were available for response and will add to above 100%

### HCP personal opinion

Results from the thematic analysis of the free text question ‘what more could be done to support those living with and beyond cancer?’ identified two key themes - current provision & support and improving existing services. Demographics of those providing free text responses can be seen in Table 6.

**Table 6:** Demographics of free text respondents

*Country of service* 100% [278]	England	81% [110]
	Scotland	9% [12]
	Northern Ireland	4% [5]
	Wales	2% [3]
*Profession* 97% [271]	Medical Oncologists	35% [48]
	Other^a^	29% [40]
	Clinical Nurse Specialists	18% [24]
	Clinical Oncologists	9% [12]
	Surgeon	6% [8]
	Medical Oncology trainee	3% [4]
	Psychologist	0% [0]
*Cancer specialty◊* 94% [262]	Breast	48% [65]
	Other (please specify)^b^	25% [34]
	Urological	24% [32]
	Lung	22% [30]
	Gynaecological	21% [29]
	Colorectal	21% [28]
	Upper Gastro-intestinal	15% [20]
	Sarcoma	12% [16]
	Hepatobiliary Nervous System	10% [13]
	Head and Neck	9% [12]
	Dermatology	7% [10]
	Lymphoma	7% [9]
	Multiple Myeloma	6% [8]
	Central Nervous System	5% [7]
	Leukaemia	3% [4]
*Institution◊* 80% [222]	Cancer Centre	52% [70]
	District Hospital	24% [33]
	Teaching Hospital	24% [33]
	General Hospital	21% [29]
	Other (please specify)^c^	4% [5]
	Primary Care	2% [2]
	Community Facility	2% [2]
*Years practicing in cancer care* 94% [262]	26+ years	19% [26]
	11–15 years	26% [35]
	16–20 years	27% [36]
	6–10 years	14% [19]
	21–25 years	12% [16]
	0–5 years	3% [4]

^a^Other answers included: radiographer, research nurse, radiotherapists, survivorship lead (project manager), occupational therapist

^b^Other answers included: across all cancers, adolescents and young adults, all radiotherapy, testicular

^c^Other answers included: private hospitals, information and support services

◊Multiple options available to respond and will add up to over 100%

#### Current provision and focus of support

Respondents detailed current support available in their service, which included, home visits, ‘cancer support charities’ clinical nurse specialists trained in CBT, holistic needs assessments, home visits, supported self-management, which included counselling, exercise support and a sexual counsellor with wellbeing events and peer support regularly available. One respondent highlighted that their service does not discharge patients, allowing a patient to access psychological support 19 years after primary treatment. However, not all care was consistent; respondents often wrote about how the current care of those leaving active treatment was akin to abandonment:

*‘Newly diagnosed patients are fully embraced with a wide range of support, but completing treatment can be like falling off a cliff into nothing’ (Cancer Nurse specialist)*

Further concerns were raised about inconsistent care both within and between trusts. Respondents indicated that support available to patients across the UK should be part of a standardised care pathway to combat the inequalities in current provision:

*‘Currently it is ad hoc and what patients may or may not be offered very much depends on where patients are treated. This wouldn’t be acceptable for treatment such as chemotherapy and shouldn’t be acceptable for after care treatment either.’ (Medical Oncologist)*

*‘I believe there are inequalities in what patients are given, even across differing heath care teams within the same cancer centre.’ (Therapeutic radiographer).*

Respondents indicated interventions should be tailored to an individual’s unmet needs and include educational, return to work, psychological (such as mindfulness training and CBT), exercise and dietary components. Respondents often stated that the focus should be on the recognition of cancer as a long term condition and helping patients resume normality.

*‘There is a need to rehabilitate patients post treatment to get them back to their pre-morbid level of health and hence increase chance of leading a normal life re return to work and the benefits that brings - financially, mentally etc.’ (Medical Oncologist)*

Family and peer support was also highlighted as an important element of the post-treatment follow-up pathway.

Respondents referred to a need for HCPs to acknowledge and address the long term effects of cancer that continue far beyond active treatment:

*‘Treating cancer is a long-term process and we have a long way to go in terms of education and raising awareness for all members of the cancer MDT, primary care, policy makers, providers, commissioners of care, the media and the public about the fact that our role as members of the cancer MDT should not finish with the end of treatment or after the 5 years of follow-up.’ (Medical Oncologist)*

Respondents described the need for more community based programmes and better linking and partnership between community teams, secondary, primary and social care as well as voluntary sectors. Further suggestions included ensuring ‘open access’ to multidisciplinary teams of ‘dieticians, psychologists, nurse specialists and Macmillan for financial advice.’

#### Improving current support services

Respondents raised frequent concerns regarding the underfunding of services, and the need for more time, staff and funding to effectively support patients living with and beyond cancer:

*‘Ongoing funding for the services is a major issue, and training of appropriate lay therapist’ (Clinical Oncologist)*

Suggested support strategies often included self-management options with end of treatment summaries with a:

‘*variety of different formats to suit more people. (ie: online, one off, peer etc…)’ (Radiographer Specialist)*

A minority of respondents stated that focusing on patients who have had cancer may take away resources from other patient groups with equal needs:

*‘In the current economic climate, there is a danger that the powerful cancer lobby will take resources away from patients with mental health issues and neurological illness which are far more debilitating in the long term than being a cancer survivor.’ (Medical Oncologist)*

## Discussion

The results of this national survey of post-cancer treatment follow-up care, represent the most up-to-date and comprehensive data-set that the authors are aware of that assesses current provision. Our key findings were that fear of recurrence, fatigue, changes in physical capabilities, anxiety and depression were highlighted by HCPs as the most important areas to address in post-treatment aftercare. There was no universal or consistent provision of any specific assessments, intervention or therapy, and the interventions offered did not clearly match views on what is most important. It is not clear how HCP priority areas of fear of recurrence, depression and anxiety are currently addressed. Psychological interventions such as CBT, which are supported by the evidence base, are amongst the least frequently offered services. Key themes from our free text analysis have indicated that provision not only varies across the country but can be inconsistent even within trusts. Rather worryingly there appears to be a gap between identified patient need and provision of care even within a cancer centre. Providing routine follow-up for patients treated with curative intent is being increasingly discouraged in the NHS in favour of patient directed self-management and therefore the ability to identify and manage patients’ needs is even more imperative. Our findings demonstrate the current variable state of provision for these patients. Knowing both which HCPs will take responsibility, how to access appropriate services and what shape those services will take is not clear. It is important to note recent data have reported that nursing and allied health care professionals involved in cancer care have expressed a need for more training around the knowledge of long-term health effects of cancer and its treatments and psychosocial care [19].

Previous prospective observational studies have reported that unmet needs at the end of cancer treatment in the NHS tend to stay unmet [4]. Since the publication of these data in 2009, it is unclear how current aftercare in the NHS has adapted to change or has improved. This is despite two key government policy documents in 2011 and 2015 highlighting the increasing importance of cancer survivorship [20, 21]. Cancer Research UK’s own report around implementation of the UK’s cancer strategies has pointed out that, whilst the importance of living with and beyond cancer services in the UK is better appreciated (likely due to growing prevalence), the 'soft’ aspect of these services can lead to them being viewed as lower priority and particularly vulnerable in ‘challenging financial climates’ [22]. Currently, such services are often not commissioned by the NHS and are left to specialist cancer charities to fund. It would appear then that a full economic evaluation of evidence-based interventions specifically for the health service would be an important contribution to this narrative.

The content and format of the interventions that are already currently offered, raise several issues. Firstly, despite an evidence base for efficacy (particularly in breast cancer) [11], approaches such as CBT were seldom available for patients. Only around a third of our sample addressed vocational or occupational rehabilitation in post-treatment aftercare. This is despite over 70% of the HCPs involved in the survey rating needs in regards to vocational/occupational problems as either important or very important. A recent systematic review of qualitative data reported that in post-treatment cancer survivors returning to vocations and paid work, this not only provides financial security but also work is an important element of self-identity, self-esteem and healthy social relationships [17]. It could be that only around a third of patients going through these services had occupations or vocations prior to cancer treatment, but that could be seen as a very conservative estimate.

Delivery of the interventions that were offered usually consisted of a one-off, face-to-face session lasting around 30–60 min. While this is much longer than a typical follow up appointment and is aimed at identifying problems and signposting patients on to further services, the effectiveness of the intervention in this format is uncertain. Such a limited investment in time may be understandable given the increasing prevalence of cancer survivors and that aftercare already struggles to fit with system capacity [22]. Even if time is limited, it is important to both match the intervention to the problem and provide an approach that has an evidence base. However, it is a matter of some uncertainty as to whether such limited contact time could reliably reproduce clinically meaningful effect sizes of interventions reported in peer-reviewed literature. For instance, this is highly unlikely to be the case in terms of fostering meaningful exercise behaviour changes and sustaining this behaviour in sedentary cancer survivors [23].

It is important to acknowledge some key limitations of this survey and analysis. The recruitment procedure did not allow us to calculate response rates for this study as professional bodies acted as intermediaries in the contact process. However, 70% of NHS acute trusts provided data. We are not aware of any other recent studies addressing these questions and as such this provides a useful and relevant available dataset on the topic. In addition, we relied on respondents to be a member of professional bodies or groups and to be actively reading emails from the group, which might have biased the sampling. Finally, we did not survey cancer related charities and voluntary agencies that also provide aftercare for cancer survivors. For example, Macmillan offer ‘The Recovery Package’ to support people living with and beyond cancer, which involves a Holistic Needs Assessment, treatment summary, cancer care review, and health and wellbeing clinics. The intervention is part of an overall self-management support package incorporating physical activity, managing the consequences of treatment, information and financial and work support [24]. Our total number of survey responders is a small proportion of the total number of NHS staff involved in cancer care.

## Conclusions

The results of our survey indicate that there does not seem to be a universal, standardised, evidence based approach to post-cancer treatment needs assessment, or interventions to either improve QoL or address unmet needs of patients. Whilst dietary and exercise advice are more frequently offered, it seems that the priority areas identified by HCP of fear of recurrence, depression and anxiety are not adequately addressed in standard practice. HCPs’ personal views highlighted that provision is often variable, not only between care centres, but even within specialities. Given the uncertain economic climate of the NHS, a full economic evaluation of an evidence based pathway would be an essential contribution to the narrative of providing cost-effective post-cancer treatment aftercare.

## Additional file

Additional file 1: SUrvivors Rehabilitation Evaluation after Cancer (SURECAN). (PDF 462 kb)

## Abbreviations

CBT: Cognitive behavioural therapy
HCP: Health care professionals
NHS: National health service
QoL: Quality of life
