# Supplementary material for: A survey to determine usual care after cancer treatment within the United Kingdom national health service
Source: BMC Cancer. 2017 Mar 11;17:186. doi: 10.1186/s12885-017-3172-1 (PMC5346235; doi:10.1186/s12885-017-3172-1)
Supplement: Additional file 1: — SUrvivors Rehabilitation Evaluation after Cancer (SURECAN). (PDF 462 kb) [file 12885_2017_3172_MOESM1_ESM.pdf]

SUrvivors Rehabilitation Evaluation after Cancer (SURECAN)

Some two million people in the UK have survived cancer, of whom a third report a diminished quality of life or wellbeing. There is a wide variation in NHS provision for these people, with unknown effectiveness and cost-effectiveness of interventions offered. The SURECAN project has as its long term aim the development of a new intervention to improve quality of life in those living with and beyond cancer.

Before an intervention to improve quality of life can be tested, we need to develop a comparison intervention based upon usual care. By filling out this short survey you will be helping us to establish what services are currently provided as part of standard follow-up care for people living with and beyond cancer within the UK. Responses are anonymous.

This survey is part of a clinical audit approved by Bart's Health NHS Trust. The survey takes an average of 10 minutes to complete. Please disregard this survey if you have previously completed the survey. If you have any questions regarding the following survey please contact one of the SURECAN research assistants:

Morvwen Duncan m.duncan@qmul.ac.uk 020 7882 8938

Jennifer Deane jennifer.deane@qmul.ac.uk 020 7882 8943

For the purpose of this survey we are interested in the care of people who have completed their active treatment aimed at a cure (eg. surgery, radiotherapy, chemotherapy) and are now living with and beyond cancer.

* 1. Where is your service based?

- England
- Scotland
- Wales
- Northern Ireland

* 2. What is your profession?

- ◯ Clinical Oncologist
- ◯ Clinical Oncology Trainee
- ◯ Clinical Nurse Specialist
- ◯ Medical Oncologist
- ◯ Medical Oncology Trainee
- ◯ Psychologist
- ◯ Surgeon
- ◯ Surgical Trainee
- ◯ Other (please specify)

* 3. What cancer specialty do you work in? (More than one option possible)

- [ ] Breast
- [ ] Colorectal
- [ ] Upper Gastro-intestinal
- [ ] Hepatobiliary Nervous System
- [ ] Dermatology
- [ ] Gynaecological
- [ ] Head and Neck
- [ ] Lung
- [ ] Leukaemia
- [ ] Lymphoma
- [ ] Multiple Myeloma
- [ ] Central Nervous System
- [ ] Sarcoma
- [ ] Urological
- [ ] Other (please specify)

* 4. How many years have you been practising within cancer care?

* 5. Approximately how many people attend your service each year who might be classed as living with and beyond cancer?

* 6.

What sort of institution do you work in? (More than one option possible)

- [ ] Cancer Centre
- [ ] Community Facility
- [ ] District Hospital
- [ ] General Hospital
- [ ] Primary Care
- [ ] Teaching Hospital
- [ ] Other (please specify)

* 7. Does your unit provide any specific assessment, interventions, or therapy for people who have completed active treatment and are living with and beyond cancer?

- ◯ Yes
- ◯ No

* 8. Within your service, what professionals are involved specifically in the care of those living with and beyond cancer? (More than one option possible)

- [ ] Clinical Oncologists
- [ ] Clinical Nurse Specialists
- [ ] Counsellors
- [ ] Psychotherapists
- [ ] Psychologists
- [ ] General Nurses
- [ ] Medical Oncologists
- [ ] Occupational Therapists
- [ ] Physiotherapists
- [ ] Surgeons
- [ ] Social Workers
- [ ] Well-being Practitioner
- [ ] None
- [ ] Other (please specify)

* 9. What are offered in your service?

	Never	Rarely	Occassionally	Frequently	Always	I don't know
Acceptance and commitment therapy	○	○	○	○	○	○
Cognitive behaviour therapy	○	○	○	○	○	○
Counselling	○	○	○	○	○	○
Dietary advice or support	○	○	○	○	○	○
Exercise therapy or advice	○	○	○	○	○	○
Family counselling/therapy	○	○	○	○	○	○
Medical assessment	○	○	○	○	○	○
Mindfulness training	○	○	○	○	○	○
One off assessment at the end of treatment	○	○	○	○	○	○
Peer support	○	○	○	○	○	○
Vocational rehabilitation/return to work programme	○	○	○	○	○	○

Other (please specify)

* 10. What aspects of living with and beyond cancer are addressed within your service?

	Never	Rarely	Occasionally	Frequently	Always
Anxiety	◯	◯	◯	◯	◯
Body image problems	◯	◯	◯	◯	◯
Changes in physical capacity	◯	◯	◯	◯	◯
Changes in cognitive capacity	◯	◯	◯	◯	◯
Depression/low mood	◯	◯	◯	◯	◯
Other emotional reactions (eg. guilt, shame, anger)	◯	◯	◯	◯	◯
Fatigue	◯	◯	◯	◯	◯
Fear of recurrence	◯	◯	◯	◯	◯
Fear of death	◯	◯	◯	◯	◯
Financial problems	◯	◯	◯	◯	◯
Low self esteem	◯	◯	◯	◯	◯
Long-term medical complications of treatment	◯	◯	◯	◯	◯
Menopausal symptoms if applicable	◯	◯	◯	◯	◯
Osteoporosis	◯	◯	◯	◯	◯
Sexual difficulties	◯	◯	◯	◯	◯
Social problems	◯	◯	◯	◯	◯
Spiritual needs	◯	◯	◯	◯	◯
Vocational/occupational problems	◯	◯	◯	◯	◯
Weight changes	◯	◯	◯	◯	◯

Other (please specify)

* 11. What service are people living with and beyond cancer offered? (More than one option possible)

- [ ] Group support facilitated by healthcare professional
- [ ] Group peer support
- [ ] Individual support facilitated by health care professionals
- [ ] Individual peer support
- [ ] None of the above (please specify)

* 12. How many sessions would a typical person living with and beyond cancer receive within your service before being discharged?

- ( ) 1
- ( ) 2
- ( ) 3
- ( ) 4
- ( ) 5
- ( ) More than 5 (please specify number)

* 13. How long is each session?

- ( ) Less than 30mins
- ( ) 30minutes - 1 hour
- ( ) 1 - 2 hours
- ( ) 2 hours +

* 14. How often do the sessions occur?

- Once
- Weekly
- Fortnightly
- Monthly
- Intermittently
- Not applicable
- Less often (please specify)

* 15. By what means are the sessions delivered? (More than one option possible)

- Telephone
- Web based
- Skype/Facetime
- Face-to-face
- Other (please specify)

Your own opinion:

We would now like you to share your own opinion on these matters.

* 16. What do you think are the most important needs of people living with and beyond cancer?

	Unimportant	Of Little Importance	Moderately Important	Important	Very Important
Anxiety					
Body image problems					
Changes in physical capacity					
Changes in cognitive capacity					
Depression/low mood					
Other emotional reactions (eg. guilt, shame, anger)					
Fatigue					
Fear of recurrence					
Fear of death					
Financial problems					
Low self esteem					
Long-term medical complications of treatment					
Menopausal symptoms if applicable					
Osteoporosis					
Sexual difficulties					
Social problems					
Spiritual needs					
Vocational/occupational problems					
Weight changes					

Other (please specify)

Your own opinion

17. What more do you think could be done to support people living with and beyond cancer?

* 18. In order to avoid more than one response from your unit, would you please state the name of your Trust or Hospital (this information will only be used to provide information where more than one clinician from the same trust has responded and not for any analysis purposes beyond this).

* 19. Would your unit be interested, in principle, in participating as a centre in a randomised controlled trial of an intervention to improve quality of life of those living with and beyond cancer? (We will not hold you to your answers; this is just to give us an indication)

○ Yes

○ No

○ Not sure

* 20. In principle, would you be willing to release one or two members of nursing or therapy staff, for three days in total, for training in the intervention and delivery of therapy? (We will not hold you to your answers; this is just to give us an indication)

○ Yes

○ No

○ Not sure

* 21. How many days could a healthcare professional be spared from your service?

* 22. In principle,would you be willing to allow the staff who have been trained to then run the therapy in their unit? (We will not hold you to your answers; this is just to give us an indication)

- Yes
- No
- Not sure

Thank you for completing this survey.

If you have any questions or comments please don't hesitate to contact one of the SURECAN research assistants:

Morvwen Duncan m.duncan@qmul.ac.uk 020 7882 8938

Jennifer Deane jennifer.deane@qmul.ac.uk 020 7882 8943
